# Supplementary material for: Karyopherin-β1 Regulates Radioresistance and Radiation-Increased Programmed Death-Ligand 1 Expression in Human Head and Neck Squamous Cell Carcinoma Cell Lines
Source: Cancers (Basel). 2020 Apr 8;12(4):908. doi: 10.3390/cancers12040908 (PMC7226044; doi:10.3390/cancers12040908)
Supplement: Supplementary file 1 [file cancers-12-00908-s001.pdf]

cancers

MDPI

Article

Karyopherin-β1 Regulates Radioresistance and Radiation-Increased Programmed Death-Ligand 1 Expression in Human Head and Neck Squamous Cell Carcinoma Cell Lines

Masaharu Hazawa, Hironori Yoshino, Yuta Nakagawa, Reina Shimizume, Keisuke Nitta, Yoshiaki Sato, Mariko Sato, Richard W. Wong and Ikuo Kashiwakura

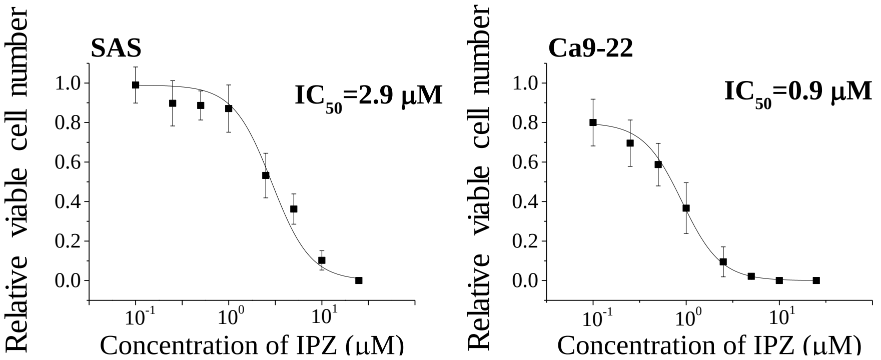

Figure S1. The effects of IPZ on the viability of HNSCC cells. SAS and Ca9-22 cells cultured in the presence of IPZ for 4 days were harvested, and the viable cell number was counted by trypan blue dye exclusion assay. The relative viable cell number compared with dimethyl sulfoxide (DMSO) control is shown. Data are presented as the mean ± SD of three independent experiments.

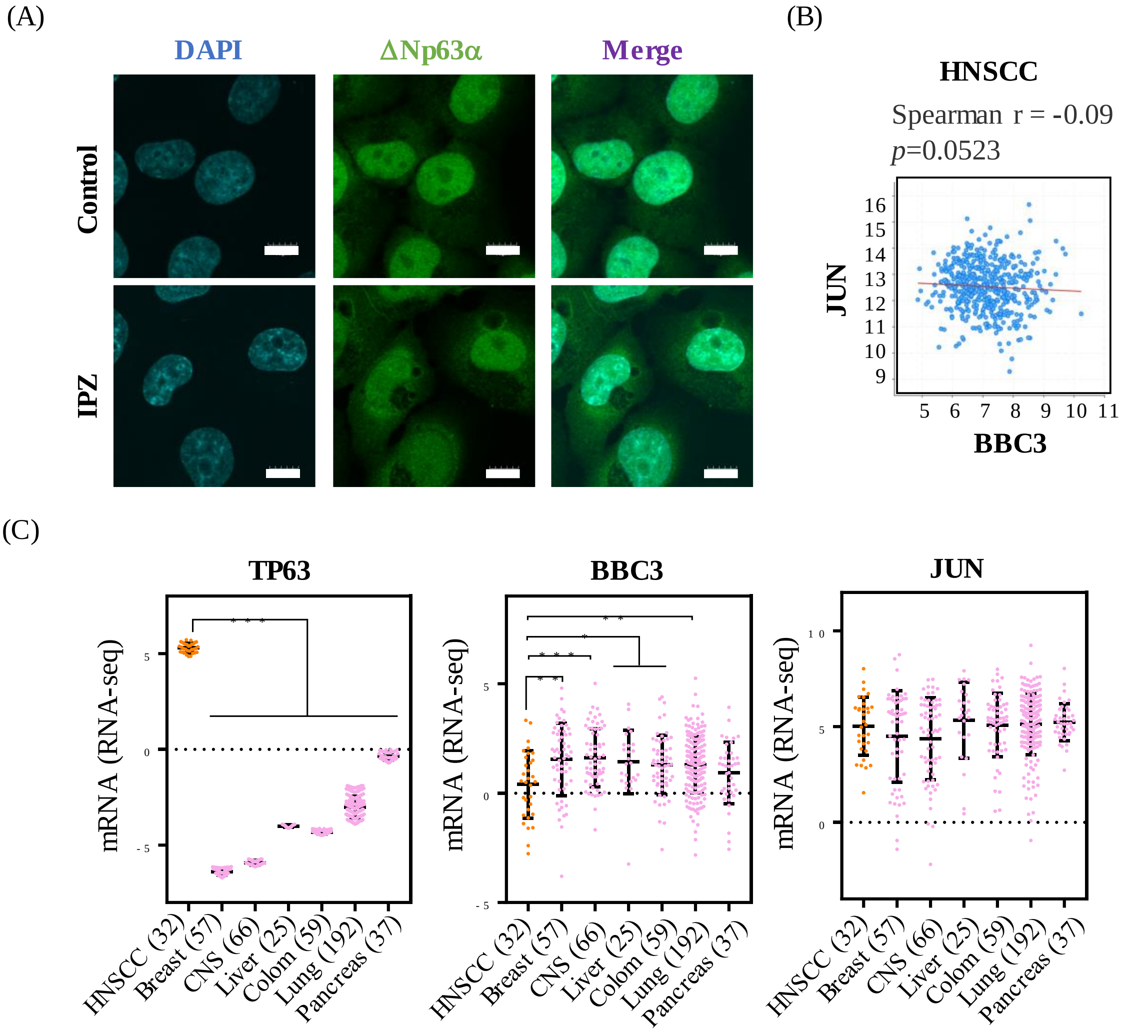

Figure S2. The analysis of relationship between BBC3 and TP63 or JUN mRNA. (**A**) Immunofluorescence confocal microscopic analysis of ΔNp63α in SAS cells after treatment with IPZ. The representative 2-D picture is shown. Bar indicates 10 μm. (**B**) The correlation between each BBC3 (PUMA) mRNA and JUN mRNA in HNSCC from TCGA. (**C**) TP63, BBC3, and JUN mRNA expression in different types of cancer cells from CCLE (http://portals.broadinstitute.org/ccle/). *, **, and *** indicate $p < 0.05$, $p < 0.01$, and $p < 0.001$, respectively.

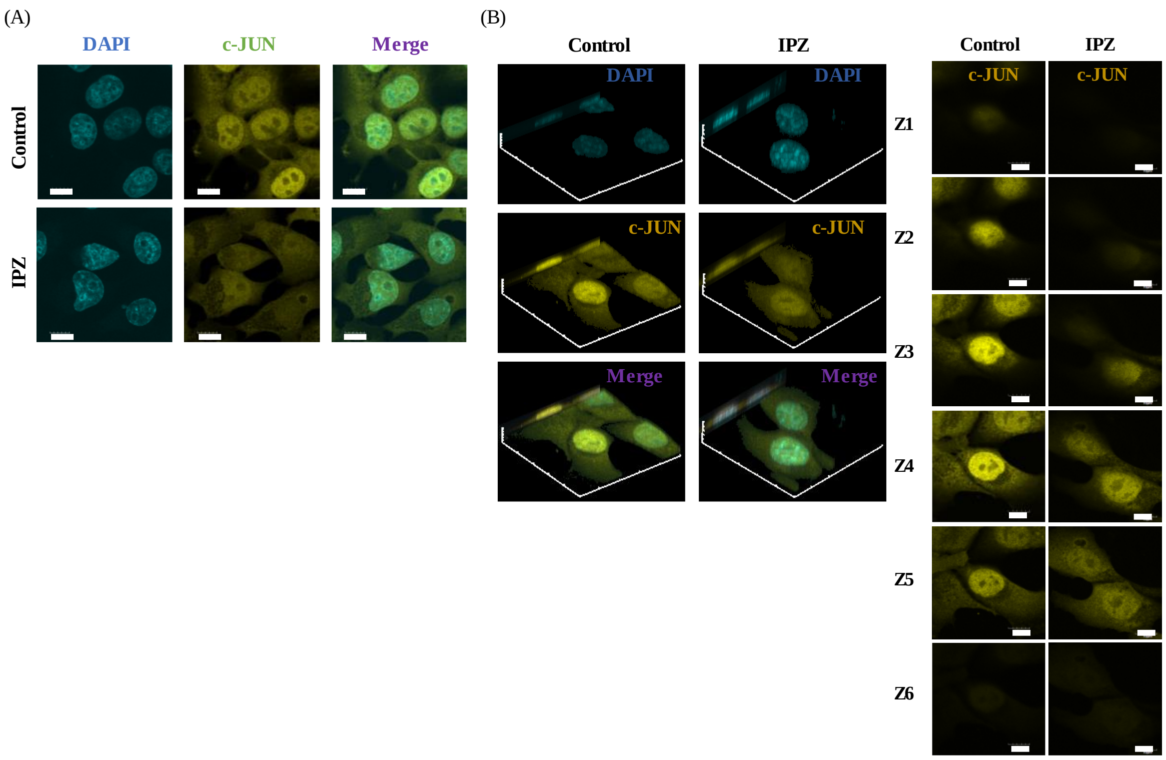

Figure S3. The effects of IPZ on nuclear localization of c-JUN in SAS cells. (A, B) Immunofluorescence confocal microscopic analysis of c-JUN in SAS cells after treatment with IPZ. Bar indicates 10 μm. (**A**) The representative two-dimensional picture. (**B**) Three-dimensional construction with maximum intensity (Left), and sequential z-stack imaging (Right) respectively.

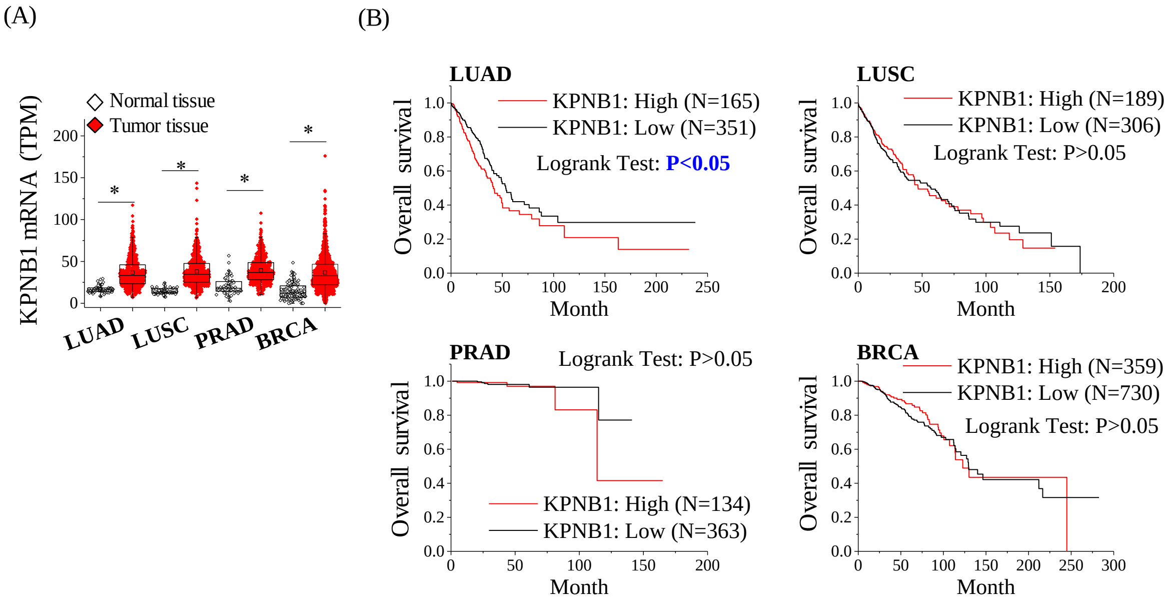

Figure S4. The relationship between KPNB1 expression and prognosis of different types of cancer patients. (**A**) The expression of KPNB1 in non-tumor tissue and lung adenocarcinoma (LUAD), lung squamous cell carcinoma (LUSC), prostate adenocarcinoma (PRAD), and breast invasive carcinoma (BRCA) samples from Cancer RNA-Seq Nexus are shown. * indicates $p < 0.01$. (**B**) The relationship between overall survival and KPNB1 expression of LUAD, LUSC, PRAD, and BRCA patients in the TCGA cohorts is shown. The patients whose KPNB1 mRNA expression z-Scores (RNA Seq V2 RSEM) is greater than 0.5 SD above mean were defined as KPNB1 high patients.

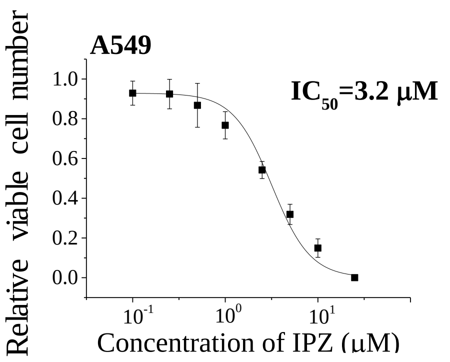

Figure S5. The effects of IPZ on cell viability of lung adenocarcinoma A549 cells. A549 cells cultured in the presence of IPZ for 4 days were harvested, and the viable cell number was counted by trypan blue dye exclusion assay. The relative viable cell number compared with DMSO control is shown. Data are presented as the mean ± SD of three independent experiments.

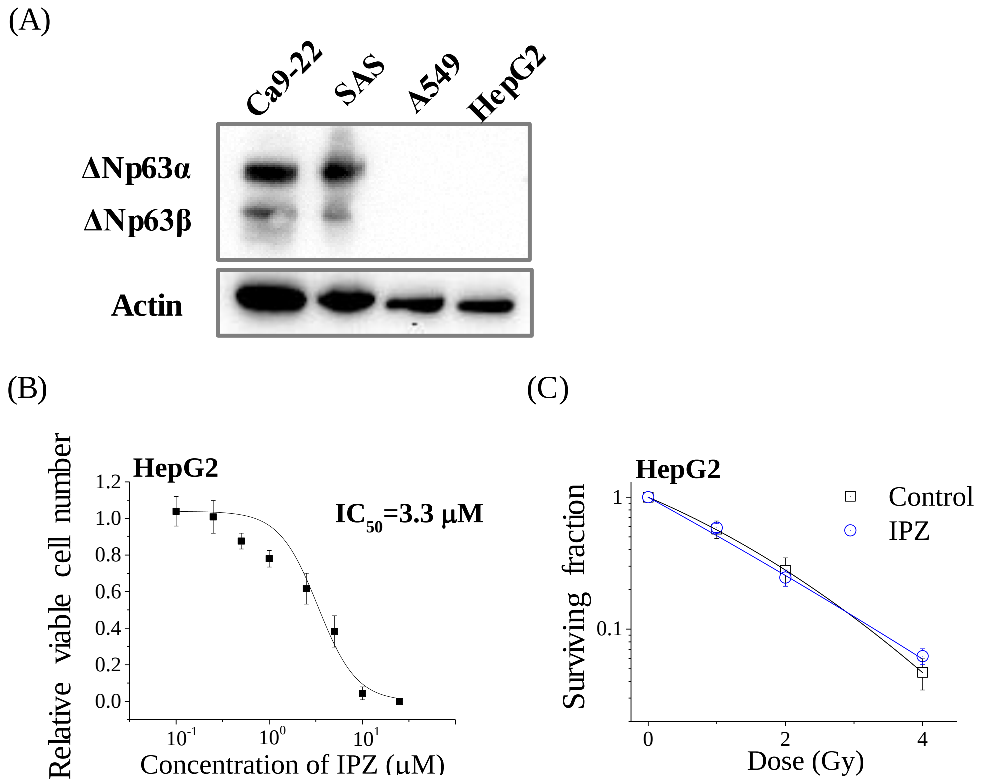

Figure S6. The effects of IPZ on proliferation and radiosensitivity of human hepatocellular carcinoma HepG2 cells. (**A**) Western blot analysis of DNp63 expression in HNSCC cells, lung adenocarcinoma A549, and hepatocellular carcinoma HepG2. The representative image is shown. (**B**) HepG2 cells cultured in the presence of IPZ for 4 days were harvested, and the viable cell number was counted by trypan blue dye exclusion assay. The relative viable cell number compared with DMSO control is shown. Data are presented as the mean ± SD of three independent experiments. (**C**) IPZ (10 μM) was added to the culture medium 1 h before X-ray irradiation. After 20 h-incubation, the cell-culture-conditioned medium was replaced with a fresh medium, and the cells were further cultured until colony was observed. The surviving fraction of HepG2 cells is shown. Data are presented as the mean ± SD of three independent experiments performed in triplicate.

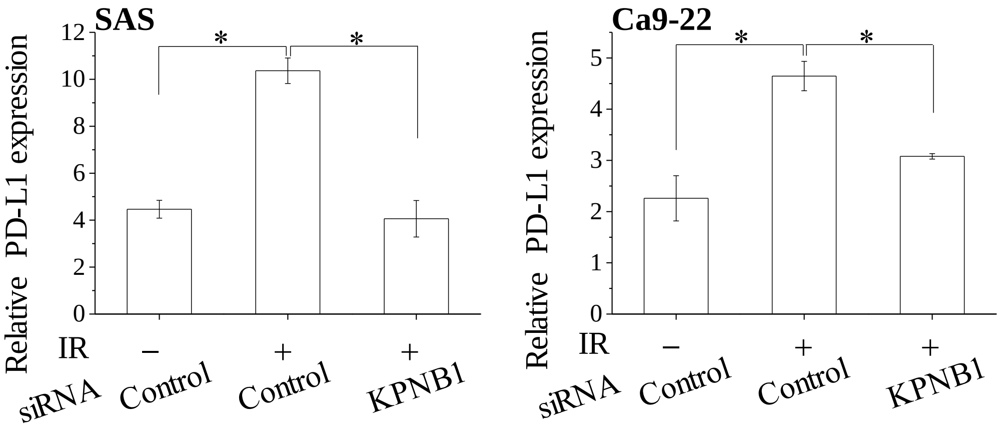

Figure S7. The effects of KPNB1 knockdown on radiation-increased cell surface PD-L1 expression on HNSCC cells. SAS and Ca9-22 cells transfected with control or KPNB1 siRNA were irradiated with 6 Gy X-ray, and cultured for 4 days. After culturing, the cells were harvested for the analysis of cell surface PD-L1 expression. The cells were harvested for the analysis of cell surface PD-L1 expression. Data are presented as the mean ± SD of three independent experiments. * indicates $p < 0.01$.

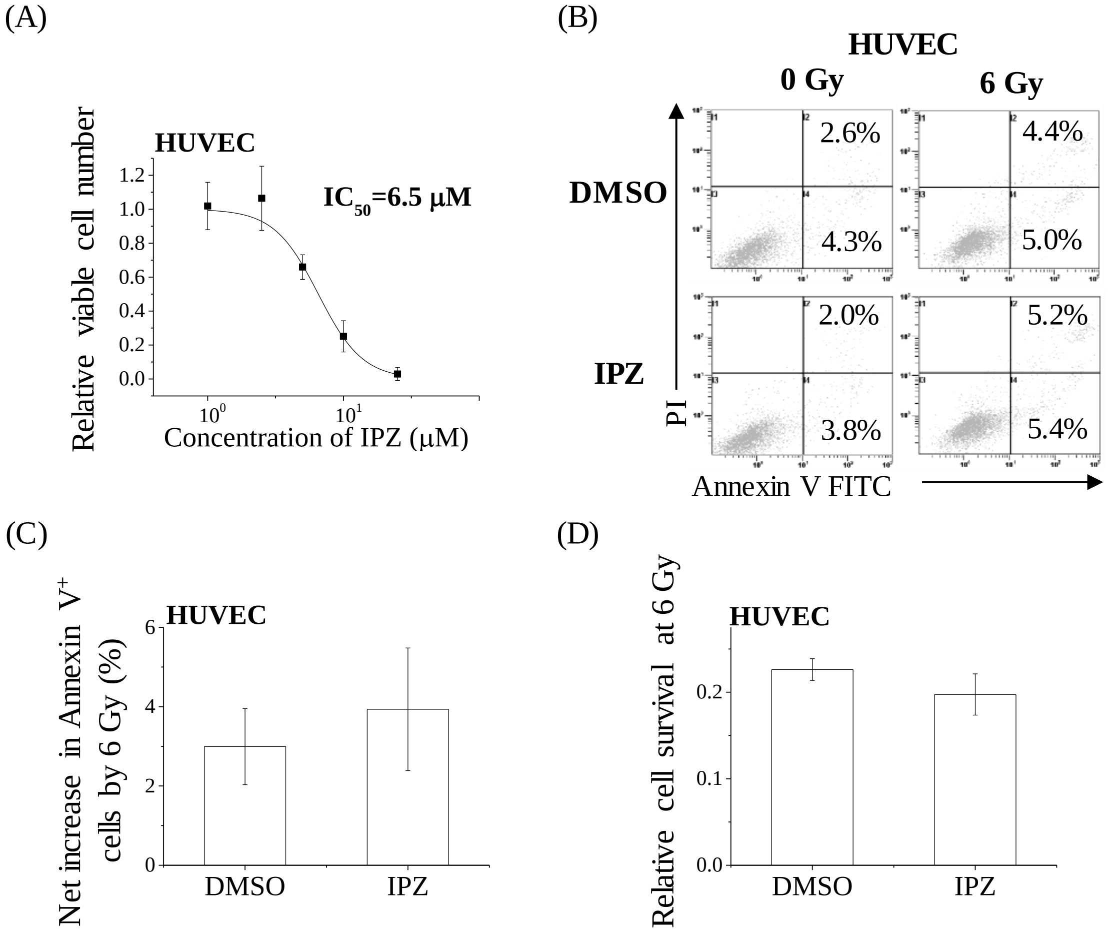

Figure S8. The effects of IPZ on proliferation and radiation-induced apoptosis of human umbilical vein endothelial cells (HUVEC). (**A**) HUVEC cultured in the presence of IPZ for 3 days were harvested, and the viable cell number was counted by trypan blue dye exclusion assay. The relative viable cell number compared with DMSO control is shown. Data are presented as the mean ± SD of three independent experiments. (**B**–**D**) IPZ (10 μM) was added to the culture medium 1 h before 6 Gy X-ray irradiation. After 20 h-incubation, the cell culture conditioned medium was replaced with a

fresh medium, and the cells were further cultured for 2 days. The cells were harvested for apoptosis assay using annexin V/PI staining. (**B**) The representative cytograms of annexin V/PI staining are shown. Inset numbers indicate the proportion of annexin V$^+$/PI$^-$ cells or annexin V$^+$/PI$^+$ cells. (**C**) Results are presented as the net increase in the population of annexin V$^+$ cells by 6 Gy irradiation. Data are presented as the mean ± SD of three independent experiments. (**D**) The relative cell survival after 6 Gy irradiation were calculated as the ratio of viable cell number of 6 Gy irradiated cells to that of non-irradiated cells. Data are presented as the mean ± SD of three independent experiments.

Supplementary information about western blot for Figure 1C, Figure 2A, Figure 3D, Figure 4A,C and Figure S6A.

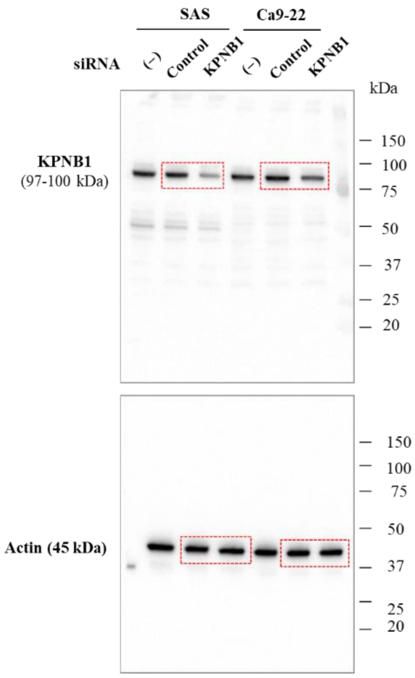

Full images of Western blotting of Figure 1(C).

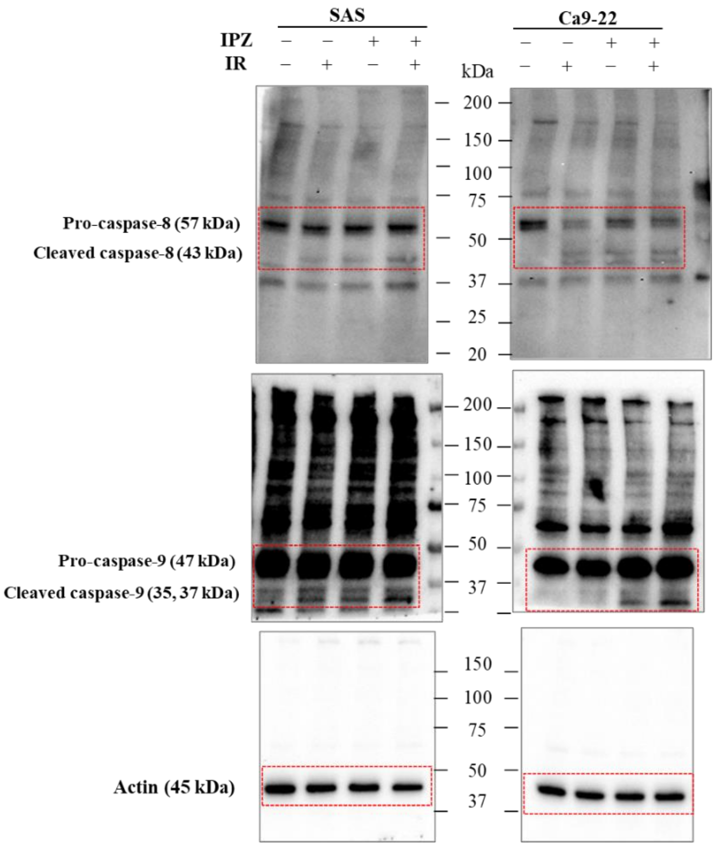

Full images of Western blotting of Figure 2(A).

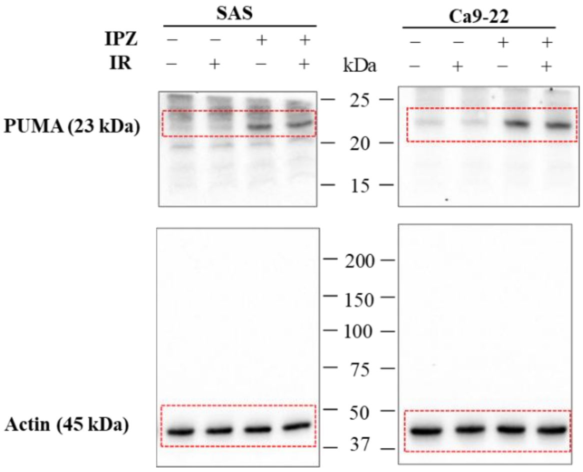

Full images of Western blotting of Figure 3(D).

Full images of Western blotting of Figure 4(A)

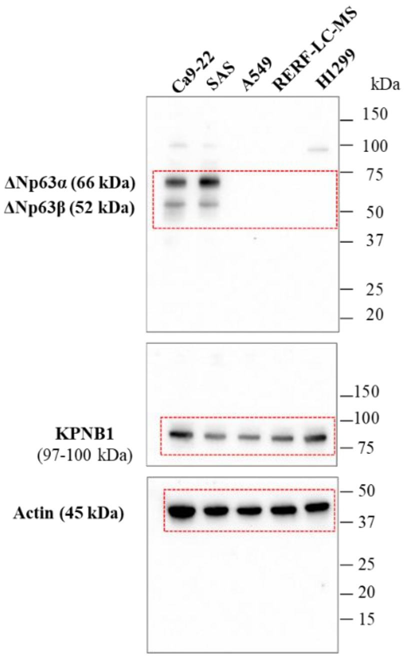

Full images of Western blotting of Figure 4(C)

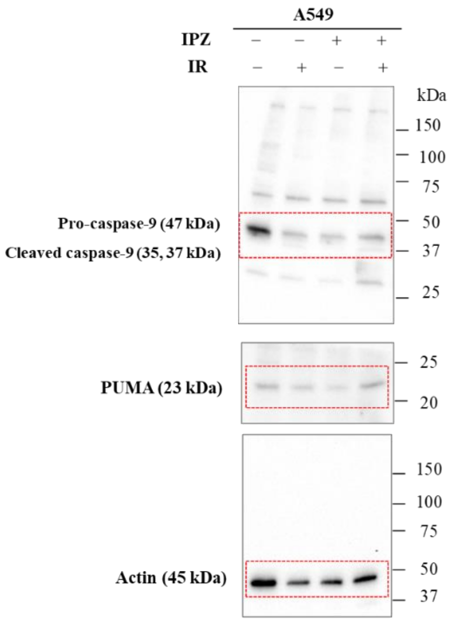

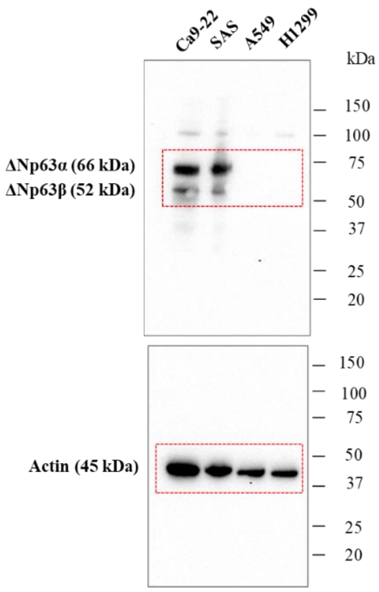

Full images of Western blotting of Figure 6(A)

© 2020 by the authors. Licensee MDPI, Basel, Switzerland. This article is an open access article distributed under the terms and conditions of the Creative Commons Attribution (CC BY) license (http://creativecommons.org/licenses/by/4.0/).
